# Point-of-care testing in the overcrowded emergency department – can it make a difference?

**DOI:** 10.1186/s13054-014-0692-9

**Published:** 2014-12-08

**Authors:** Kevin D Rooney, Ulf Martin Schilling

**Affiliations:** Intensive Care Unit, Royal Alexandra Hospital and University of the West of Scotland, Corsebar Road, Paisley, PA2 9PN UK; Center of Clinical and Experimental Medicine, University of Linköping and Department of Accidents and Emergencies, Linköping University Hospital, Lasarettgatan 1, 581 85 Linköping, Sweden

## Abstract

Emergency departments (EDs) face several challenges in maintaining consistent quality care in the face of steadily increasing public demand. Improvements in the survival rate of critically ill patients in the ED are directly related to the advancement of early recognition and treatment. Frequent episodes of overcrowding and prolonged waiting times force EDs to operate beyond their capacity and threaten to impact upon patient care. The objectives of this review are as follows: (a) to establish overcrowding as a threat to patient outcomes, person-centered care, and public safety in the ED; (b) to describe scenarios in which point-of-care testing (POCT) has been found to ameliorate factors thought to contribute to overcrowding; and (c) to discuss how POCT can be used directly, and indirectly, to expedite patient care and improve outcomes. Various studies have shown that overcrowding in the ED has profound effects on operational efficiency and patient care. Several reports have quantified overcrowding in the ED and have described a relationship between heightened periods of overcrowding and delays in treatment, increased incidence of adverse events, and an even greater probability of mortality. In certain scenarios, POCT has been found to increase the number of patients discharged in a timely manner, expedite triage of urgent but non-emergency patients, and decrease delays to treatment initiation. This review concludes that POCT, when used effectively, may alleviate the negative impacts of overcrowding on the safety, effectiveness, and person-centeredness of care in the ED.

## Introduction

Emergency departments (EDs) face a number of important challenges in the modern health-care environment. Simultaneously confronted with decreasing hospital resources and growing public demand, EDs frequently experience prolonged waiting times and extended periods of overcrowding. Minimizing the delay between the onset of symptoms and the initiation of therapy is critical to improving outcomes for critically ill patients. Overcrowding forces EDs to operate beyond their capacity and commonly results in delays in diagnosis and treatment, potentially impacting upon patient care. The impact of overcrowding on public health and quality of care in the ED has received national attention, prompting urgent calls for reform [1]. Consequently, there exists a critical and urgent need for methods to enhance patient flow in order to lessen the burden of overcrowding in the ED and to improve the overall quality of emergency care.

Point-of-care testing (POCT) provides physicians with rapid results for many commonly ordered tests. The implementation of POCT in an ED setting has been suggested as a means to increase timely discharge rates, shorten length of stay, and increase patient throughput. When used in appropriate scenarios, POCT could be an effective tool to minimize the time-to-treatment initiation and to improve patient outcomes. This review will discuss the problem of overcrowding, stressing its impact on patient safety and care. Relevant examples from the literature will be discussed, highlighting specific scenarios in which POCT has been shown to expedite patient care and to impact patient outcomes positively. As with any new tool, identifying the opportunities in which that tool can be most effectively used is a critical first step.

## Review

### Overcrowding in the emergency department

Overcrowding is a global problem with a multitude of negative consequences on efficiency and quality of care [2]. Metrics of overcrowding and quality indicators in the ED community vary greatly. Common definitions include an increase in waiting and processing time, a lack of bed capacity in the ED, a general perception of being rushed by emergency physicians and staff, increased ambulance diversions, and increased frequency of patients leaving the ED without being seen [3].

Overcrowding can have a significant impact on the timeliness and quality of care in the ED. Prolonged waiting times and overcrowding have been associated with substantial delays in the administration of antibiotics [4] and pain medication [5]. Several studies have reported a relationship between overcrowding in the ED and mortality [6,7]. One retrospective study examined data from three metropolitan hospitals over the course of a 3-year period in Australia [7]. Using high hospital and bed occupancy rates in the ED to quantify periods of overcrowding, the researchers evaluated the impact of overcrowding on the incidence of patient mortality on days 2, 7, and 30. The results show a significant linear relationship between overcrowding and mortality, and an estimated 120 deaths per year are hypothesized to be associated with overcrowding. Another retrospective cohort study evaluated data from all EDs in the greater Ontario area over the course of a 5-year period [6]. Here, length of stay in the ED was used to define overcrowding. A significantly greater risk of death was found with increasing length of stay in the ED. The overall severity of these effects on patient outcomes suggests that overcrowding can justifiably be regarded as issues of public health and safety rather than simply a problem of ED efficiency.

A commonly cited reason for overcrowding is the boarding of admitted patients in the ED when hospital beds are unavailable [8]. Financial pressures make a significant increase in hospital bed capacity as a solution to overcrowding unlikely. Changes to front-end operations and optimizing existing clinical pathways suggest a more practicable approach. For example, ensuring an efficient and rapid triage service can efficiently help to reduce overcrowding in the ED [9]. Increasing the speed at which low-risk and non-emergent patients are identified will reduce downstream burdens, freeing up time for emergency physicians to spend on more critically ill or time-sensitive patients. Additionally, the rapid identification of high-risk patients will decrease delays in treatment initiation, potentially improving outcomes and shortening overall length of stay.

### Point-of-care testing

POCT refers to any diagnostic test administered outside the central laboratory at or near the location of the patient. In the past, the size and complexity of equipment required to perform medical testing required a centralized hospital laboratory. With advances in technology, it has become increasingly possible to perform common clinical investigations outside of the laboratory at the point of care with a reasonable level of accuracy (Table 1). The primary advantages provided by POCT devices are increased portability and speed. Using POCT, caregivers can perform, analyze, obtain, and act on test results at the bedside in a matter of minutes, significantly faster than if samples were sent out to a central laboratory. If used effectively, POCT has the potential to decrease delays to treatment initiation, increase ED efficiency, influence patient care positively, and alleviate the negative effects of overcrowding.

**Table 1:** **Sensitivity and specificity of selected point-of-care analysis compared with core-laboratory analysis**

**Marker**	**Sensitivity, %**		**Specificity, %**		**Reference**
	**POCT**	**Core laboratory**	**POCT**	**Core laboratory**	
CKMB + cTnT					
Single draw	30	30	91	92	[10]
Serial draw	43	43	88	91	
hCG (urine)	95.3	100	100	100	[11]
hCG (blood)	95.8	100	100	100	
D-dimer	83.3	100	100	100	[12]
	100	100	73.3	67.9	[13]

CKMB, creatine kinase-MB; cTnT, cardiac troponin T; hCG, human chorionic gonadotropin; POCT, point-of-care testing.

Numerous reports highlight decreases in turnaround times (TATs) for test results with POCT in an emergency setting [10,14]. One study compared TATs between POCT and laboratory testing when a tube transport system was implemented for the rapid transport of samples. Even under circumstances that sought to minimize sample transit times, POCT results were available an average of 46 minutes earlier than from the central laboratory [15]. Several studies evaluating point-of-care pregnancy testing in the ED found that, regardless of whether ED staff evaluated pregnancy status by using urine or a qualitative human chorionic gonadotropin immunoassay kit, POCT could yield sufficiently sensitive results faster than if samples were sent to a central laboratory [11] as processing and handling delays inherently extended laboratory TATs.

Current literature shows that the real-life impact of POCT in the ED can vary greatly. The magnitude of the effect POCT has on patient care and on efficiency shows a strong dependency on the clinical context. Rapid TATs for test results are most beneficial in cases in which (a) delays in treatment of at least 1 hour can have significant effects on outcomes and (b) delays in test results are the primary determining factor holding up patient management decisions. Interestingly, recent evidence suggests that POCT can add value when used at the pre-hospital level [16-18]. The following sections examine several instances in which evidence supports the advantages of implementing POCT in EDs.

### Acute coronary syndrome

Patients presenting with symptoms of acute coronary syndrome (ACS) represent a sizable proportion of total attendees in the ED. Approximately 70% of patients who are admitted to the ED with suspicion of ACS are later ruled out after further investigation [19]. Conversely, a non-trivial number of patients with ACS are mistakenly discharged home from the ED, resulting in avoidable patient mortality. Clearly, a means of rapidly and accurately assessing risk status in suspected patients with ACS would benefit efficiency of the ED, patient care, and outcomes.

Biochemical evaluation of ACS with cardiac biomarkers, specifically cardiac troponins (Tns), is becoming increasingly common [20] and has proven to be a sensitive indicator of myocardial injury [21]. Current guidelines suggest that Tn measurements be made available to physicians within 30 minutes of sample collection [20], and delays in treatment are commonly associated with an increased probability of adverse outcomes [22,23]. Disruptions in laboratory processing times during periods of overcrowding can negatively impact patient care and further strain ED resources. Several available POCT technologies exist for cardiac TnI testing with TATs of not more than 20 minutes [24,25]. In one report, the time delay between blood draw and physician review was 42.1 minutes shorter when POCT was used, compared with samples that were sent to a central laboratory [26]. A rapid rule-out protocol using a combination of high-sensitivity cardiac TnI testing, risk score, and electrocardiogram was recently shown to be safe and effective in identifying low-risk patients [27].

POCT has been shown to increase the speed at which positive cases of ACS are accurately identified [16,28]. Importantly, these gains in diagnostic speed have been found to translate into decreased times to percutaneous coronary intervention and related treatment initiation [14,28]. A number of studies have documented the impact of cardiac biomarker POCT on measures of ED efficiency. The rapid TATs provided by POCT have universally been found to reduce the average length of stay in the ED or hospital [29,30]. A UK multicenter randomized controlled study evaluated the performance of POCT for cardiac biomarkers on patients with suspected myocardial infarction [31]. The researchers showed a 20% greater discharge rate during the initial evaluation process when POCT was performed. However, a follow-up study evaluating the financial implications of POCT found variable results [32]. A separate study evaluating the use of B-type natriuretic peptide in patients presenting with dyspnea found substantial cost savings with POCT [33], suggesting that the impact on overall costs can vary greatly among institutions. Although there is little direct evidence that POCT in ACS results in superior outcomes [34], an argument can be made that increased efficiency in the ED will indirectly benefit patient care by alleviating the negative effects of overcrowding.

### Venous thromboembolic disease

Venous thromboembolic disease (VTD), including pulmonary embolism and deep venous thrombosis, can be a serious and potentially life-threatening condition. The mortality rates in patients with pulmonary embolism can be high, yet symptoms are often mild and easy to miss [35]. Therefore, the availability of methods for the accurate identification of patients with suspected VTD is critically important.

Risk assessment for VTD first divides patients into low-risk and high-risk subgroups. The large majority (~70%) of patients fall into the low-risk category and often are tested for D-dimer, a fibrin degradation product formed as a result of fibrinolysis or clot degradation. It has been shown that, in patients with a Wells score of not more than 4, a negative D-dimer result can be used to safely rule out deep venous thrombosis, eliminating the need to waste time and resources on further testing [36,37]. Various POCT devices exist for D-dimer with negative predictive values higher than 98% [38]. Evaluation of these devices in an ED setting found pronounced effects on TATs and measures of efficiency. One study evaluated the effect of POCT on the time to D-dimer results from triage in an emergency setting [12]. The researchers found a median difference of 101.5 minutes between groups, with POCT providing significantly faster results than traditional laboratory testing. Another study compared performance of the ED before and after implementation of a whole-blood rapid D-dimer POCT device [13]. Researchers found that, following POCT, mean length of stay in the ED decreased by approximately 1 hour and hospital admission rates dropped by 14%.

### Severe sepsis

Sepsis is a life-threatening disease that arises when the body’s response to infection injures its own organs and tissue. Despite advances in vaccines, antibiotics, and critical care, sepsis remains the primary cause of death from infection and is one of the leading causes of death in the world [39]. The clinical signs and symptoms of sepsis (tachycardia, tachypnea, and pyrexia) are generally very non-specific, making it difficult to identify patients with sepsis at an earlier stage and to treat them in a timely manner. Delays in treatment can have a profound negative effect on outcomes. Therefore, an accurate and immediate means of identifying patients with severe sepsis is critically important for minimizing delays to resuscitation and maximizing clinical benefits.

**Figure 1:**
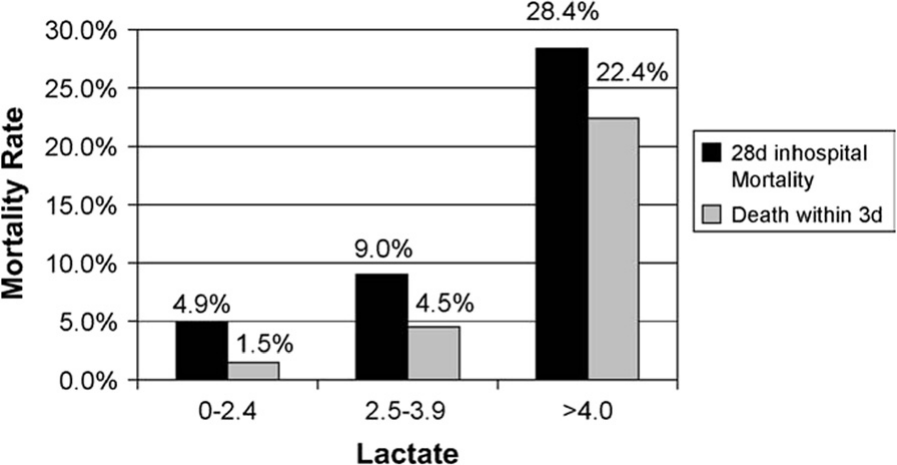
**Serum lactate as a predictor of mortality in emergency department (ED) patients with sepsis.** In-hospital mortality rates were documented for patients who presented to the ED with infection, and serum lactate measurements were available (n = 1,278). Greater 3-day and 28-day mortality rates were found in patients with lactate levels of more than 4.0 mmol/L. Reprinted with permission from Elsevier [46].

Elevated blood lactate levels have been shown to be a sensitive marker of impaired tissue perfusion and of anaerobic metabolism in patients with suspected sepsis, predictive of mortality, and are a valid identification method for patients who will benefit from early aggressive goal-directed therapy [40]. Recent guidelines suggest immediate targeted fluid resuscitation as a means of normalizing lactate in these patients [41]. Rapid measurement of lactate is important as elevated lactate levels have been linked to increased mortality, with a sizable percentage of deaths occurring within the first few days (Figure 1). The initiation of goal-directed therapy within the first 3 to 6 hours of presentation to the ED has been found to improve mortality rates by 16% [41,42]. Additionally, decreases in lactate levels of 20% every 2 hours for the first 8 hours have been associated with a 9.6% reduction in mortality [43]. Delays in triage and test processing times because of overcrowding have the potential to significantly delay test results and increase time-to-treatment initiation. Valid POCT technologies for measuring whole blood and fingertip lactate are available, providing almost immediate feedback. One study found that POCT for fingertip lactate during triage was associated with results being available to physicians 151 minutes earlier than when whole-blood lactate levels were taken at the discretion of the treating physician [44]. This study did not evaluate medical outcomes, but a separate study found improved outcomes in patients with higher lactate clearance at 6 hours compared with patients with lower clearance [45]. This relationship between time-to-treatment initiation and patient outcome highlights the need for expedited diagnosis through POCT.

### Stroke

During recent years, thrombolysis has revolutionized the treatment of ischemic stroke, increasing long-term survival and reducing morbidity. Owing to an increased risk of bleeding with delayed thrombolysis, minimizing delays in treatment is crucial in the modern treatment of non-hemorrhagic stroke. Reducing delays between symptom onset and initiation of thrombolysis has been shown to be a critical factor in predicting positive outcomes and reducing overall mortality rates [47]. The immediate assessment of a patient’s coagulation status (that is, international normalized ratio, partial thromboplastin time, hemoglobin, and platelet count) is necessary to ensure timely and safe thrombolysis. Early computed tomography (CT) scan, CT angiography, and diffusion-weighted imaging scanning are also needed in order to exclude hemorrhagic stroke and to elucidate the potential benefit of thrombolysis. Delays in laboratory results were recently identified as one of the major barriers to the early initiation of thrombolysis therapy in a simulation-based approach [48], highlighting a need for improved efficiency in testing. Several studies have clearly shown the benefits of POCT in the management of stroke. Results show that POCT can reduce TATs by 30 to 50 minutes and can significantly increase the incidence of early thrombolysis, positively impacting patient care [18,49].

### Challenges to effective implementation of point-of-care testing

Important challenges exist with any significant change to existing ED methodologies. Clinical pathways and ED logistics may need substantial modification to maximize the clinical and economic benefits of rapid TATs provided by POCT. Furthermore, the direct cost per analysis for the majority of POCT devices is higher than the cost per analysis in centralized laboratories. In this clinical context and with respect to the numerous manual steps to be performed in transferring a blood sample to the central laboratory and to retrieve the results consecutively, the total costs of POCT devices tend not to exceed those of central analysis. Clinical trials in academic hospitals did not reveal clear disadvantages of POCT versus central analysis, whereas trials in rural regions were in favor of POCT [31,50].

Most POCT devices are operated by staff with limited technical background. Cooperation from central laboratory personnel is therefore essential to ensure adequate quality of test results. Additionally, POCT places additional responsibilities on ED nurses, the staff members who most commonly perform POCT. Nurses must undergo regular training and meet certification requirements for quality assurance purposes, placing additional burdens on individuals with an already heavy workload. On the other hand, expediting patient flow through POCT might help to reduce this strain on staff. Additional oversight and regulatory challenges will exist for any substantial implementation of POCT in the ED. These costs must be weighed against the potential gains in efficiency and in patient care that one can realistically expect to gain from POCT.

These challenges can be met via internal quality control and external quality assessment (EQA) methods. Internal quality control methods help ensure that devices are producing accurate and consistent results by analyzing the output of a control sample. Device manufacturers may provide the necessary control materials, or other control samples can be used. Likewise, many devices offer internal quality control functionality and may require quality control checks to be performed and documented before releasing patient results. If the POCT device has network access, then quality control measurements can be integrated into a central data management system. EQA uses samples containing an unknown-to-the-operator value of reagent received from an external source, such as from an accredited EQA program or device manufacturer. Additionally, the manpower challenges imposed by POCT can largely be offset through indirect efficiency benefits from improved patient care. A rapid turnover of non-critical patients will help to reduce overcrowding, and a lower incidence of critical or deteriorating patients in the ED will free up costly time that nurses and staff would otherwise spend attending to these high-risk patients.

## Conclusions

Overcrowding represents a serious impediment to the ability of the ED to provide the public with quality emergency care. Prolonged waiting times and treatment delays can have substantial effects on patient satisfaction and outcomes. Increased mortality rates in the ED suggest that overcrowding should be treated as a serious public health concern and not solely as a problem of departmental efficiency. Being of multifactorial origin, overcrowding should be seen as a problem of hospital-wide patient flow and could be influenced by improved diagnostic protocols. Rapid TATs from POCT represent one means by which clinical decision making and patient management might be expedited to counteract several of the negative effects of overcrowding on ED performance. When used effectively and in the appropriate context, POCT has been shown to reduce delays to treatment initiation in the critically ill, improve outcomes, increase timely patient discharge rates, and decrease total length of stay. Elevated costs of POCT per analysis seem to be outweighed by the total gain of expedited patient flow in the appropriate setting. Continual advances in POCT promise to broaden the applications of this technology and provide further opportunities to improve the quality of pre-hospital and hospital emergency care. Developments in comprehensive POCT technology for the complete blood count, pregnancy testing, infectious diseases, and cancer screening promise to change the way emergency medicine is practiced. As technology advances and POCT devices continue to expand their test menus and functionality, it seems only a matter of when, and not whether, POCT becomes commonplace in emergency medicine.

## Abbreviations

ACS: Acute coronary syndrome
CT: Computed tomography
ED: Emergency department
EQA: External quality assessment
POCT: Point-of-care testing
TAT: Turnaround time
Tn: Troponin
VTD: Venous thromboembolic disease
